# Optimizing the number of players and training bout durations in soccer small‐sided games: Effects on mood balance and technical performance

**DOI:** 10.1002/ejsc.12208

**Published:** 2025-02-18

**Authors:** Zouhaier Farhani, Hatem Ghouili, Wissem Dhahbi, Achraf Ammar, Mohamed Ben Aissa, Mohamed Mansour Bouzouraa, Khaled Trabelsi, Noomen Guelmami, Nizar Souissi, Ismail Dergaa, Anissa Bouassida, Lamia Ben Ezzeddine

**Affiliations:** ^1^ Sport Sciences Health and Movement (3SM) High Institute of Sport and Physical Education of Kef University of Jendouba Kef Tunisia; ^2^ High Institute of Sport and Physical Education of Ksar‐Said University of Manouba Manouba Tunisia; ^3^ Physical Activity Research Unit Sport and Health (UR18JS01) National Observatory of Sports Manouba Tunisia; ^4^ Department of Training and Movement Science Institute of Sport Science Johannes Gutenberg‐University Mainz Mainz Germany; ^5^ Research Laboratory Molecular Bases of Human Pathology LR19ES13 Faculty of Medicine of Sfax University of Sfax Sfax Tunisia; ^6^ High Institute of Sport and Physical Education Sfax University of Sfax Sfax Tunisia; ^7^ Interdisciplinary Laboratory in Neurosciences Physiology and Psychology: Physical Activity Health and Learning (LINP2) UFR STAPS (Faculty of Sport Sciences) UPL Paris Nanterre University Nanterre France; ^8^ Department of Human and Social Sciences High Institute of Sport and Physical Education of Kef University of Jendouba Kef Tunisia; ^9^ Department of Health Sciences (DISSAL) Postgraduate School of Public Health University of Genoa Genoa Italy; ^10^ Primary Health Care Corporation (PHCC) Doha Qatar

**Keywords:** football, physiology, psychology, recovery, team sport, training

## Abstract

This study aimed to determine the effects of different bout durations (1 × 12 min, 2 × 6 min and 3 × 4 min) of three‐a‐side (3vs3) and four‐a‐side (4vs4) small sided games (SSGs) with goalkeepers, on the profile of mood state (POMS) scores, and technical performance (percentage of successful passes, percentage of successful tackles, percentage of successful duels, and percentage of ball loss) in soccer players. Methods: Sixteen semiprofessional male soccer players participated in the study (age: 20.7 ± 0.7 years, height: 179.5 ± 6.1 cm, body mass: 67.2 ± 4.9 kg, body fat: 10.7 ± 0.7%). In randomized counterbalanced order, participants completed the six different conditioned SSGs (2 playing‐formats × 3 bout‐durations). POMS scores and technical performance data were collected during each bout of SSGs. Results: The data demonstrated that the continuous‐bout‐duration (1 × 12 min) of 4vs4 and 3vs3 SSGs was characterized by a significant decrease in total mood disturbance (TMD) compared to 2 × 6 min and 3 × 4 min (4vs4:*p* < 0.01; 3vs3:*p* < 0.001). Continuous bout duration showed a greater (*p* < 0.05) percentage of successful passes compared to 2 × 6 min (4vs4; *d* = 2.57 [very large] and 3vs3: *d* = 1.79 [large]) and 3 × 4 min (4vs4: *d* = 2.14 [very large] and 3vs3; *d* = 1.73 [large]). The percentage of successful tackles was only greater (*p* < 0.05) for 1 × 12 min in 4vs4 (2 × 6 min; *d* = 0.83 [moderate] and 3 × 4 min: *d* = 0.86 [moderate]) and successful duels in 3vs3 (2 × 6 min; *d* = 1.41 [large] and 3 × 4 min; *d* = 1.43 [large]). Conclusion: The bout durations in four‐ and three‐a‐side soccer games seem to influence behavioral and technical performance of the players. Therefore, coaches should consider longer continuous bouts when planning SSGs‐based training to significantly decrease TMD and enhance technical‐tactical performance in soccer SSGs.

## Highlights

- Mood Enhancement: Continuous 12‐min bouts in 4vs4 and 3vs3 small‐sided games significantly reduce total mood disturbance (TMD) compared to shorter durations (2 × 6 min and 3 × 4 min).
- Technical Precision: Continuous bouts exhibit a significantly higher percentage of successful passes, tackles, and duels, emphasizing the importance of bout duration in enhancing technical‐tactical performance.
- Strategic Coaching: Coaches should prioritize continuous bouts in four‐ and three‐a‐side soccer games for improved mood balance and enhanced technical‐tactical performance.

## 1 INTRODUCTION

Small‐sided games (SSGs) involve playing soccer games in reduced spaces with fewer than 11 players, such as 3vs3 or 4vs4 (Chen et al., 2023; Farhani et al., 2022). In the context of soccer, these games serve as multifunctional training exercises that allow soccer coaches and fitness coachs to utilize various forms of SSGs during training. This approach aims to enhance physical, technical, tactical, and psychological skills simultaneously (Aguiar et al., 2015; Gonçalves et al., 2016; Guard et al., 2022). By simulating match environments, these games effectively develop the skills and abilities of soccer players (Hill‐Haas et al., 2010). The adoption of SSGs during regular soccer training serves as a significant motivator and fosters a more developed psychological state in players compared to other forms of athletic soccer training (Los Arcos et al., 2015; Selmi et al., 2017).

Additionally, SSGs can effectively test and develop individual and group emotional and social mental skills. They also contribute to better motivation, a more positive state of mind, and help maintain the training load of the players (Köklü et al., 2017; Selmi et al., 2020). This is achieved by fostering team spirit and competition while controlling risk‐taking and actions (Silva et al., 2014). It is important to note that the number of players and the duration of each bout can impact the training load and psychological state of players (Aslan, 2013; Esqueda et al., 2022; López‐Fernández et al., 2018). Specifically, the number of players and the duration of small‐sided game sessions can influence the training load. Indeed, significant differences have been reported in the responses to the drills for each intensity measure (Little et al., 2007). Both heart rate (HR) and rate of perceived exertion (RPE) tend to decrease as the number of players in the drill increases (Little et al., 2007). Moreover, these factors can lead to physical boredom and psychological tiredness, affecting the concentration and mood of the players (Clemente et al., 2021; Freitas et al., 2014).

Given the multitude of factors involved and the importance of SSG training, it is crucial to develop a comprehensive understanding of its physical, physiological, and psychological effects. This understanding forms the foundation for effectively managing and adapting the training load to accommodate the various modes of SSGs (Clemente et al., 2021; Freitas et al., 2014; Little et al., 2007). Extensive research has unequivocally shown that modifying the constraints within SSGs environments significantly affects the demands of the activity and the resulting physiological responses (Foster et al., 2010; Hill‐Haas et al., 2011). For example, Foster et al. showed a significant effect of the number of players on the increase in HR in 4 versus 4 SSGs than in 6 versus 6 SSGs (Foster et al., 2010; Hill‐Haas et al., 2011). As a result, it is imperative for the technical staff of teams to possess the necessary knowledge and expertise to meticulously structure training loads based on SSGs (Mallo et al., 2008). Previous studies have explored various aspects of SSGs, such as exercise type, boot selection, duration, and the number of players. These studies have focused on how these variables affect physical and physiological performance. Research indicates that altering the number of players in SSGs can change the peak heart rate (HR peak) in soccer players. Adjusting these parameters can also help target higher training loads. Furthermore, a greater number of players is associated with lower approximate entropy values, suggesting better positional organization in SSGs with more participants (Aguiar et al., 2015; Dellal, 2020; Guard et al., 2022). However, we were unable to find large‐scale studies that have attempted to demonstrate the effect of bout duration and the number of players during SSGs on the mood state and technical performance in soccer players.

Considering that sports performance in soccer depends on various factors, we have decided to investigate the impact of manipulating the duration of SSG sessions and the number of players on the psychological aspect and technical skills of soccer players. Therefore, the main objective of this study was to assess the influence of different bout durations and the number of soccer players, including goalkeepers, during SSGs, on mood balance and technical performance.

## 2 MATERIALS AND METHODS

### 2.1 Study design

The study was conducted over 5 weeks (2 weeks were dedicated to the anthropometric measurements and the yo‐yo test as well as to familiarize the participants with the testing procedures and the formats of the SSGs and 3 weeks for evaluation) during the mid‐season and utilized a quasi‐experimental design with a repeated measures approach. The same player performed 3 SSGs conditions in a randomly counterbalanced order: (a) 1 bout of 12 minutes (1 × 12 min), (b) 2 bouts of 6 min (2 × 6 min), and (c) 3 bouts of 4 min (3 × 4 min) on different days simultaneously for 3 weeks at the same time (between 14:00 and 15:00), and on the same synthetic grass pitch.

### 2.2 Participants

Sixteen semiprofessional male soccer players (age: 20.7 ± 0.7 years, height: 179.5 ± 6.1 cm, body mass: 67.2 4.9 ± kg, body fat: 10.7 ± 0.7%, soccer‐playing experience: 6.9 1.2 ± years) volunteered to participate and finished all the experimentation process in this study. These players competed for the same high‐level team in the national league in Tunisia, training 5–6 days per week (7.5 ± 1.54 h of training/week) with one official competition (i.e., match) per week. To ensure the accuracy of our study, it was essential that all participants were fully committed to attending exercise sessions. Throughout the entire study, none of the soccer players suffered any injuries. For the final analysis, we only considered data from players who demonstrated unwavering dedication to their exercise routine, achieving a perfect adherence rate of 100%. To maintain consistency, we excluded individuals who used dietary supplements during the study period or engaged in non‐team training activities. Goalkeepers were also excluded from the analysis. After applying these criteria, we concluded the study with 16 players only.

### 2.3 Procedures

The present study began during a mid‐season and spread over five weeks of which two weeks are devoted to familiarization and three weeks for experimental interventions. Before and immediately after each SSGs, all players filled in the mood questionnaire (POMS), and technical analysis data collection was performed, using video cameras during each session of SSGs. During the familiarization phase with the experimentation protocol, participants proceeded to undertake the yo‐yo intermittent recovery Test (YIRT) level 1. Their performance in the YIRT determined their ranking, which was based on the maximal distance covered. In order to ensure a fair distribution of skills and fitness levels within the SSGs teams, the coach also assessed each player's overall technical and tactical skill level using a 5‐point Likert scale ranging from 1 (poor) to 5 (excellent). This evaluation aimed to prevent any disparities between skill and fitness levels. The technique used for assigning players to groups, known as minimization, is thoroughly explained in previous expert literature (Altman et al., 2005). It is important to note that the talent selection should be based on a combination of objectively measured data and coaches' assessments (Sieghartsleitner et al., 2019). We also recorded anthropometric measures during this period.

The SSGs protocol involved games with teams of 4 versus 4 and 3 versus 3 on pitches sized 25 × 32 m and 30 × 20 m, respectively. The relative pitch size was set at 100 m^2^, and the recovery time between SSGs bouts established at 2 min (Köklü et al., 2017). SSGs were performed with the goalkeeper but were not included in the study analysis. Supervision and verbal encouragement were continuously provided by the coaches, and spare balls were available to each team whenever the ball was out of bounds or when a goal was scored. The characteristics of the SSGs (number of bouts, bout duration (min), and pitch dimensions [length × width] (m^2^), recovery time (min), and rest type) are shown in Table 1. Each SSGs was preceded by a 20‐min specific warm‐up, which consisted of low‐intensity jogging, striding, coordination movements, and dynamic stretching (Selmi et al., 2020). Players were allowed to consume ad libitum water during the recovery periods.

**TABLE 1:** Characteristics of SSGs formats employed.

Formats	Game structures	Duration (min)	Total playing area (m2)	Relative playing area (m2)	Recovery between bouts (min)^a^	Goalkeepers
4vs4	Continuous	1 × 12	32 × 25	100	2	Yes
	Medium	2 × 6				
	Small	3 × 4				
3vs3	Continuous	1 × 12	30 × 20	100	2	Yes
	Medium	2 × 6				
	Small	3 × 4				

Abbreviations: SSGs, small‐sided games; 4vs4, 4 players + 1 goalkeeper Versus 4 players + 1 goalkeeper; 3vs3, 3 players + 1 goalkeeper Versus 3 players + 1 goalkeepers.

^a^ Passive recovery.

### 2.4 Yo‐yo intermittent recovery test (YIRT)

The YIRT was utilized to assess the participants' maximal aerobic endurance fitness (Bangsbo et al., 2008) and to gain insights into their overall fitness levels. This test involved a series of 20‐m shuttle runs with participants running back and forth between the starting, turning, and finishing lines until they reached a point of exhaustion. To ensure consistency and control, the test incorporated audio bleeps from a recording, which gradually increased the running speed. The initial speed level was set at 10 km·h‐1. The test was concluded when a participant could no longer maintain the required running speed for two consecutive occasions as indicated by the bleep or if they felt unable to complete the stage. The test was conducted on a synthetic grass field with groups of six players participating simultaneously. Throughout the test, the participants' heart rates were closely monitored, and the highest heart rate measurement (HRpeak) was recorded.

### 2.5 The profile of mood state (POMS)

Mood state was measured using the French–Canadian version of the POMS developed by Shacham (1983) and translated by Fillion and Gagnon (Fillion et al., 1999). The inventory contains 37 items rated on a 5‐point Likert scale (0 indicates “Not at all” and 4 indicates “extremely”) in responses to questions such as “How are you feeling right now?” and includes six subscales: Tension‐anxiety, Depression‐dejection, Anger‐hostility, Vigor‐activity, Fatigue‐inertia, and Confusion‐bewilderment. The total mood disturbance (TMD) was calculated as follows: [TMD = ((Anger + Confusion + Depression + Fatigue + Tension) − Vigor)) + 100] (Shacham, 1983). All players completed the POMS questionnaire 15 minutes before the SSGs and immediately at the end of the SSGs. The questionnaire was administered to players individually. The Cronbach's *α* coefficients ranged from 0.83 to 0.91, indicating high internal consistency.

### 2.6 Small‐sided games

The SSGs consisting of 4‐a‐side and 3‐a‐side teams were played on two different pitches. The first pitch measured 25 × 32 m2, while the second pitch measured 20 × 30 m2. The goals for both games were 3 m in length and 2 m in height. The relative pitch size, which is the ratio of the playing area to the number of players, was set at 100 m2/player for both the 4‐a‐side and 3‐a‐side games. This decision was based on previous expert literature and took into consideration that changing the pitch size would affect the training load and the technical‐tactical performance (Clemente et al., 2021; Olthof et al., 2018). The duration of the SSGs varied depending on the format: (a) one bout of 12 minutes (1 × 12 min), (b) two bouts of six minutes (2 × 6 min), and (c) three bouts of four minutes (3 × 4 min). A two‐minute passive recovery period was allowed between each bout of the intermittent SSGs. To ensure the smooth flow of the game, coaches encouraged the players and provided new balls when necessary. This allowed for uninterrupted play during the exercises.

### 2.7 Video technical analysis

The SSGs were recorded using video cameras (Canon HFG40, Canon Inc). These cameras provided unrestricted access to the entire field, allowing the observer to pause, rewind, or replay the footage as needed. This feature enabled the observer to accurately count and assess all the technical parameters being investigated. To evaluate the players' performance, the percentage of successful passes, duels, tackles, and ball loss were analyzed based on the total number of actions performed by each player. These variables have been previously defined and studied in research focusing on the technical and tactical aspects of soccer players (Praça et al., 2015; Yi et al., 2020). A successful pass was defined as a pass that reached its intended target without being intercepted by an opponent. Duels involved direct competitions between opposing players with the objective of gaining possession of the ball. Successful tackles were recognized as the defender's legal act of dispossessing an opponent who was in possession of the ball. Lastly, the ball loss percentage referred to the number of possessions lost out of the total number of times a player touched the ball. It is important to note that goals were not taken into consideration in this analysis, as the goalkeepers were not classified according to their skill level. Based on previous research, the SSGs videos were analyzed twice by the same qualified researcher (sports scientist specialized in soccer) to test the reliability of the analysis system (Sieghartsleitner et al., 2019).

### 2.8 Statistical analysis

All statistical analyses were conducted using IBM SPSS Statistics for Windows, version 28.0 (IBM Corp). The data is presented as mean ± standard deviation (SD). A significance level of *p* ≤ 0.05 was uniformly established as the cut‐off criterion for statistical significance in this study. The normality of the distribution for all variables was assessed using the Kolmogorov–Smirnov test, and it was found that all dependent variables followed a normal Gaussian distribution. Therefore, a repeated measure analysis of variance (RMANOVA) was conducted to determine the significance of differences SSGs on two within‐subject factors: bout duration and number of players. The dependent variables examined were POMS and technical performance. To assess mood state responses, a RMANOVA was used with three within‐subject factors: number of players (4vs4 and 3vs3), SSGs duration (12 min, 2 sets of 6 min, or 3 sets of 4 min), and effort (pre‐ and post‐exercise session). The interactions between these factors were also examined on the scores of the six subscales and the total mood disturbance (TMD).

When a significant interaction effect was found, the analysis was completed with a post hoc Bonferroni test. The effect size was evaluated with eta partial squared (ƞ2*p*), where ƞ2*p* values less than 0.06 were regarded as indicative of a small effect, values ranging from 0.06 to less than 0.14 were categorized as moderate, and values equal to or exceeding 0.14 signified a large effect (Hopkins et al., 2009). The magnitude of the differences for the post hoc comparisons was calculated as Cohen's d with Hedges' corrections (Lakens, 2013). As per the categorization established by Hopkins et al. (2009), d was delineated into the following classifications: ‘trivial’ (<0.2), ‘small’ (>0.2–0.6), ‘moderate’ (>0.6–1.2), ‘large’ (>1.2–2), or ‘very large’ (>2) (Hopkins et al., 2009). When interpreting the presented differences, it is important to consider that a conservative correction Bonferroni was used for the statistical comparisons. Additionally, given the considerably large number of comparisons, it is crucial to pay special attention to the effect sizes and confidence intervals. This will help avoid relying solely on *p*‐values and prevent type‐I errors (Greenland, 2019; Rothman, 2010).

## 3 RESULTS

### 3.1 Mood state

The RMANOVA for mood state variables revealed significant interactions between Bout‐duration × Number‐of‐Players × Time on the majority of POMS variable except the TMD score (*p* = 0.2) and confusion (*p* = 0.87) (Table 2). Significant interactions between bout duration *x* number of players and bout duration × time were found in the majority of the tested variables except confusion (*p* = 0.7 and 0.8, respectively). There was no significant interaction between Number‐of‐Players × Time in any of the tested parameters (*p* > 0.5). As shown in Table 2, significant main effects of Bout‐duration, Number‐of‐Players, and Time were found for the majority of tested parameters except vigor. Post hoc results are detailed in Table 3. Significant differences from before (T0) to after (T1) SSGs sessions were found in all POMS variable except vigor for the 1 × 12 min bout duration using both 4vs4 and 3vs3 formats. For the 2 × 6 min bout duration, these significant changes were only registered for depression, confusion, and anger using both formats and TMD only, using the 4vs4 format (*p* = 0.07). No significant changes were recorded in any of the tested variables for the 3 × 4 min bout duration.

**TABLE 2:** Bout duration, number of players’ acute SSGs exercise’ effects on POMS.

Variables		Main effects			Interactions			
		Bout duration	Number of players	Time	Bout duration × number of players	Bout duration × time	Number of players × time	Bout duration × number of players × time
TMD	(AU)	F(2.60) = 52.12 *p* = 0.001 ƞ2*p* = 0.63	F(1.30) = 28.14 *p* = 0.001 ƞ2*p* = 0.48	F(1.30) = 17.95 *p* = 0.001 ƞ2*p* = 0.37	F(2,60) = 3.07 *p* = 0.05 ƞ2*p* = 0.09	F(2.60) = 7.52 *p* = 0.001 ƞ2*p* = 0.2	F(1,30) = 0.19 *p* = 0.66 ƞ2*p* = 0.006	F(2,60) = 1.5 *p* = 0.2 ƞ2*p* = 0.04
Fatigue	(AU)	F(2.60) = 7.76 *p* = 0.001 ƞ2*p* = 0.2	F(1.30) = 69.75 *p* = 0.001 ƞ2*p* = 0.69	F(1.30) = 29.84 *p* = 0.001 ƞ2*p* = 0.49	F(2,60) = 3.46 *p* = 0.03 ƞ2*p* = 0.1	F(2.60) = 50.22 *p* = 0.001 ƞ2*p* = 0.62	F(1,30) = 0.68 *p* = 0.79 ƞ2*p* = 0.002	F(2,60) = 3.34 *p* = 0.04 ƞ2*p* = 0.1
Confusion	(AU)	F(2.60) = 23.76 *p* = 0.001 ƞ2*p* = 0.43	F(1.30) = 6.9 *p* = 0.01 ƞ2*p* = 0.18	F(1.30) = 23.78 *p* = 0.001 ƞ2*p* = 0.25	F(2,60) = 0.33 *p* = 0.7 ƞ2*p* = 0.01	F(2.60) = 0.15 *p* = 0.8 ƞ2*p* = 0.005	F(1,30) = 0.006 *p* = 0.93 ƞ2*p* = 0.0001	F(2,60) = 0.13 *p* = 0.87 ƞ2*p* = 0.005
Depression	(AU)	F(2.60) = 3.56 *p* = 0.003 ƞ2*p* = 0.1	F(1.30) = 4.7 *p* = 0.03 ƞ2*p* = 0.13	F(1.30) = 7.92 *p* = 0.009 ƞ2*p* = 0.2	F(2,60) = 22.22 *p* = 0.0001 ƞ2*p* = 0.42	F(2,60) = 45.22 *p* = 0.001 ƞ2*p* = 0.6	F(1,30) = 1.07 *p* = 0.3 ƞ2*p* = 0.03	F(2,60) = 38.01 *p* = 0.001 ƞ2*p* = 0.55
Vigor	(AU)	F(2.60) = 2.23 *p* = 0.1 ƞ2*p* = 0.06	F(1.30) = 1.41 *p* = 0.2 ƞ2*p* = 0.04	F(1.30) = 0.55 *p* = 0.4 ƞ2*p* = 0.01	F(2,60) = 3.22 *p* = 0.04 ƞ2*p* = 0.09	F(2.60) = 1.01 *p* = 0.3 ƞ2*p* = 0.03	F(1,30) = 0.16 *p* = 0.9 ƞ2*p* = 0.001	F(2,60) = 9.7 *p* = 0.001 ƞ2*p* = 0.24
Anger	(AU)	F(2.60) = 42.36 *p* = 0.001 ƞ2*p* = 0.58	F(1.30) = 29.48 *p* = 0.001 ƞ2*p* = 0.49	F(1.30) = 19.15 *p* = 0.001 ƞ2*p* = 0.39	F(2,60) = 3.08 *p* = 0.05 ƞ2*p* = 0.09	F(2.60) = 9 *p* = 0.001 ƞ2*p* = 0.23	F(1,30) = 0.09 *p* = 0.7 ƞ2*p* = 0.003	F(2,60) = 5.24 *p* = 0.008 ƞ2*p* = 0.14
Tension	(AU)	F(2.60) = 31.65 *p* = 0.001 ƞ2*p* = 0.51	F(1.30) = 0.008 *p* = 0.9 ƞ2*p* = 0.001	F(1.30) = 29.81 *p* = 0.001 ƞ2*p* = 0.49	F(2,60) = 2.26 *p* = 0.1 ƞ2*p* = 0.07	F(2.60) = 3.47 *p* = 0.03 ƞ2*p* = 0.1	F(1,30) = 0.26 *p* = 0.8 ƞ2*p* = 0.001	F(2,60) = 4.04 *p* = 0.02 ƞ2*p* = 0.11

Abbreviations: AU, arbitrary units; POMS, Profile of Mood States test; SSGs, small sided games; TMD, total mood disorder score; ƞ2*p*, Effect size.

**TABLE 3:** Effects of bout duration, number of players, and times on POMS.

Variables	Formats	Bout: 1 × 12 min				Bout: 2 × 6 min				Bout: 3 × 4 min			
		T0	T1	׀d׀	*P*	T0	T1	׀d׀	*p*	T0	T1	׀d׀	*p*
Depression (AU)	4vs4	11 ± 1.2	9 ± 0.9	2 ± 1.2	<0.001***	9.69 ± 1.4	8.81 ± 1.1	0.87 ± 0.1	0.01**	9.19 ± 1.5	8.69 ± 1.4	0.5 ± 1.3	0.3
	3vs3	9 ± 0.9	7.81 ± 1.1	1.18 ± 1.04	0.002**	7.68 ± 0.6	6.87 ± 0.5	0.81 ± 0.7	0.02*	11.5 ± 1.8	11.12 ± 0.9	0.37 ± 2.2	0.4
Fatigue (AU)	4vs4	7.18 ± 2.3	9.31 ± 1.4	−2.12 ± 1.7	<0.001***	7.46 ± 1.9	8 ± 1.7	−0.5 ± 0.8	0.29	5 ± 0.8	5.3 ± 0.9	−0.31 ± 0.9	0.32
	3vs3	8.93 ± 1.2	11.31 ± 1.2	−2.37 ± 0.8	<0.001***	7.81 ± 1.04	8.6 ± 1.08	−0.81 ± 1.4	0.13	7.25 ± 0.6	7.75 ± 1.18	−0.5 ± 1.21	0.11
Confusion (AU)	4vs4	9.37 ± 2.8	7.25 ± 2.5	2.12 ± 1.7	0.006**	8.93 ± 1.2	8.18 ± 0.9	0.75 ± 1.4	0.06*	8.12 ± 1.62	7.56 ± 1.7	0.56 ± 1.8	0.3
	3vs3	10.31 ± 1.1	8.31 ± 1.35	2 ± 0.9	0.009**	9.5 ± 1.2	8.68 ± 1.3	0.81 ± 1.8	0.04*	9.12 ± 1.8	8.43 ± 1.54	0.68 ± 0.9	0.5
Tension (AU)	4vs4	11.18 ± 3.03	8.93 ± 2.8	2.25 ± 1.9	0.007**	10.25 ± 1.9	9.56 ± 1.1	0.68 ± 1.15	0.1	9.75 ± 1.29	9.12 ± 1.2	0. 62 ± 0.8	0.4
	3vs3	12.25 ± 1.3	10.12 ± 1.14	2.12 ± 1.08	0.01*	10.43 ± 1.6	9.81 ± 0.98	0.62 ± 1.54	0.2	8.43 ± 1.2	7.87 ± 1.02	0.56 ± 0.7	0.1
Anger (AU)	4vs4	10.93 ± 0.9	8 ± 1.3	2.93 ± 1.5	<0.001***	7.68 ± 2.1	6.43 ± 0.8	1.25 ± 1.7	0.02*	8.37 ± 2.12	8 ± 2.3	0.37 ± 1.9	0.6
	3vs3	11.43 ± 1.4	8.43 ± 0.9	3 ± 1.15	<0.001***	10.37 ± 1.7	9.18 ± 1.3	1.18 ± 2.3	0.03*	9.37 ± 1.4	8.81 ± 1.3	0.56 ± 0.5	0.3
Vigor (AU)	4vs4	11.62 ± 0.8	11.18 ± 0.4	0.43 ± 0.8	0.3	12.81 ± 0.9	12.43 ± 1.2	0.37 ± 1.5	0.4	12.5 ± 0.8	12.25 ± 1.06	0.25 ± 0.77	0.5
	3vs3	12.68 ± 1.8	12.18 ± 0.75	0.5 ± 1.89	0.2	11.93 ± 1.4	11.68 ± 1.3	0.25 ± 1.43	0.5	11.62 ± 0.9	11.43 ± 1.5	0.18 ± 1.8	0.6
TMD (AU)	4vs4	138 ± 7.7	131 ± 6.8	6.75 ± 4.1	0.001***	131.18 ± 59	128 ± 3.2	2.62 ± 3.9	0.07	127.9 ± 3.5	126.43 ± 3.5	1.6 ± 3.6	0.18
	3vs3	139.25 ± 3.4	133.81 ± 3.16	5.43 ± 3.68	0.008**	133.87 ± 4.25	131 ± 2.03	2.37 ± 4.14	0.1	134.06 ± 2.3	132.56 ± 3.3	1.5 ± 2.8	0.18

Abbreviations: AU, arbitrary units; POMS, Profile of Mood States test; SSGs, small sided games; TMD, total mood disorder score; T0, Before training; T1, After training; ׀d׀, difference between before and after session of SSGs.

* Significant differences from before (T0) and after (T1) SSGs sessions for each bout duration, at *p* < 0.05.

** Significant differences from before (T0) and after (T1) SSGs sessions for each bout duration, at *p* < 0.01.

*** Significant differences from before (T0) and after (T1) SSGs sessions for each bout duration, at *p* < 0.001.

### 3.2 Technical‐tactical performance

**FIGURE 1:**
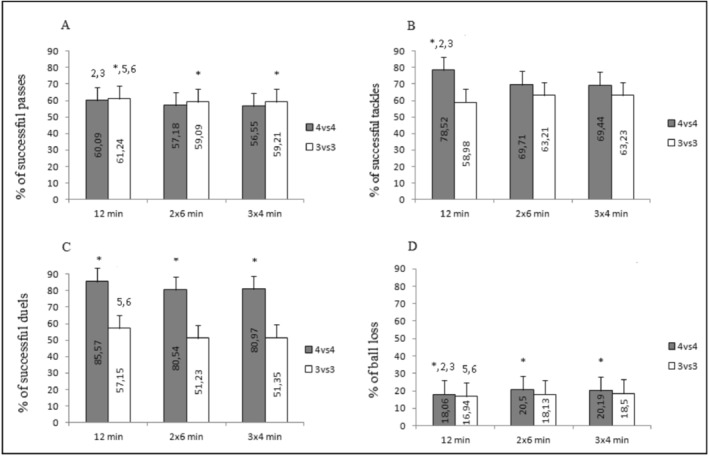
Mean values ± SD of technical‐tactical performance during 4vs4 and 3vs3 soccer small‐sided games (SSGs) at different bout durations (1 × 12 min, 2 × 6 min, and 3 × 4 min).

Results presented in Figure 1 showed a significant interaction between bout duration and the number of players on the % of successful passes (F(2, 30) = 3.83, *p* = 0.03, ƞ2*p* = 0.20[large]), tackles (F(2, 30) = 5.35, *p* = 0.01, ƞ2*p* = 0.26[large]), and ball loss (F(2,30) = 4.54, *p* = 0.02, ƞ2*p* = 0.23[large]). As a result, the interaction between the within‐subject factors was not significant for the % of successful duels (F (2, 30) = 0.20, *p* = 0.82, ƞ2*p* = 0.01[small]). Considering that both factors independently caused an effect on this variable (bout duration: F(2, 30) = 12.28, *p* < 0.001; ƞ2*p* = 0.45[large]; number of participants: F(1,15) = 1195.59, *p* < 0.001, ƞ2*p* = 0.99[large]).

Bonferroni post hoc comparisons presented in Figure 1 revealed that the continuous bout duration (1 × 12 min) was characterized by a significantly greater percentage of successful passes compared to 2 × 6 min (4vs4: *p* < 0.001, *d* = 2.57 [very large]; 3vs3: *p* < 0.001, *d* = 1.79 [large]) and 3 × 4 min (4vs4: *p* < 0.001, *d* = 2.14 [very large]; 3vs3: *p* < 0.001, *d* = 1.73 [large]), and minor ball loss rate also compared to 2 × 6 min (4vs4: *p* < 0.001, *d* = 2.14 [very large]; 3vs3: *p* = 0.006, *d* = 1.11 [moderate]) and 3 × 4 min (4vs4: *p* < 0.001, *d* = 2.11 [very large]; 3vs3: *p* < 0.001, *d* = 1.57 [large]). The percentage of successful tackles was only significantly greater for 1 × 12 min in 4vs4 (comparison with 2 × 6 min: *p* = 0.05, *d* = 0.83 [moderate]; comparison with 3 × 4 min: *p* = 0.05, *d* = 0.86 [moderate]) and the percentage of successful duels in 3vs3 (comparison with 2 × 6 min: *p* = 0.001, *d* = 1.41 [large]; comparison with 3 × 4 min: *p* < 0.001, *d* = 1.43 [large]).

A, B, and C represent the percentages of successful passes, tackles, and duels, respectively, while D represents the percentage of lost balls.

*: Statistically significant difference (*p* < 0.05) between 4vs4 and 3vs3; 1, 2, 3, 4, 5, 6: statistically significant difference with conditions.

1: Condition 1 (12 min/4vs4); 2: Condition 2 (2 × 6 min/4vs4); 3: Condition 3 (3 × 4 min/4vs4); 4: Condition 4 (1 × 12 min/3vs3); 5: Condition 5 (2 × 6 min/3vs3); and 6: Condition 6 (3 × 4 min/3vs3), respectively.

## 4 DISCUSSION

The purpose of this study was to investigate how the duration of bouts (1 × 12 min, 2 × 6 min, and 3 × 4 min) and the number of players (4vs4 and 3vs3) affect mood state variables and technical performance during soccer small‐sided games (SSGs).

The major finding of this study revealed that longer bout duration (1 × 12 min) positively influences total mood disturbance and technical performance. These results highlight the importance for coaches to consider these variables and ensure that SSGs are properly designed to align with the training objectives for soccer players while also assessing internal and external stresses. By doing so, coaches can promote the development of practical skills, reduce the risk of overtraining, and optimize the benefits of SSGs within their overall training plans. Based on our findings, the duration of 4vs4 and 3vs3 SSGs had a notable impact on various emotional states. Specifically, both continuous (i.e., 1 × 12 min) and intermittent (2 × 6 min) bout durations resulted in a significant decrease in depression, anger, tension, confusion, and total mood disturbance (TMD). However, the effect on vigor was not statistically significant. It is worth noting that fatigue scores increased in both formats (4vs4 and 3vs3). This can be explained by the fact that the intense training load elicits an increase in fatigue and decreased level of force for players. Likewise, the stability of vigor can be explained by the fact that SSGs training caused mental fatigue for players and caused mood disturbance related to higher intensities, which associated with unpleasant sensations. These results suggest that longer durations of SSGs can have a positive influence on the emotional well‐being of soccer players. By engaging in extended periods of gameplay, players experience a reduction in negative emotions and an overall improvement in mood. This finding highlights the potential benefits of incorporating longer SSGs into training sessions for soccer players.

To the best of our knowledge, few studies have explored the connection between mood and the duration of SSGs in soccer. One of the most noteworthy findings of this study is that negative emotions such as depression, anger, tension, fatigue, and confusion were significantly lower during the continuous duration (1 × 12 min) and the medium intermittent duration (2 × 6 min) compared to the short intermittent duration (3 × 4 min) in both the 4vs4 and 3vs3 formats. This suggests that SSGs have the ability to enhance motivation and enjoyment over extended periods while also improving players' concentration, enabling them to execute a greater number of successful passes and maintain ball possession, thus facilitating effective attacking and marking strategies (Carraro et al., 2014; Farhani et al., 2022). These conditions, however, also imply an increase in the mental load and psychological state of players (Selmi et al., 2017). In a similar vein, Casamichana and Castellano (Casamichana et al., 2010) demonstrated that playing for shorter durations could lead to physiological and mood disturbances among soccer players.

Oliveira et al. (2013) found that continuous training sessions resulted in more positive emotional responses compared to high‐intensity interval training sessions, albeit with increased tension and decreased vigor. Our study extends these findings by demonstrating that continuous 12‐min bouts in SSGs significantly reduced total mood disturbance (TMD) and improved technical performance compared to shorter durations.

While Oliveira et al. (2013) suggested reducing games to 4vs4 for four episodes of 4 min to maintain mood stability, our results indicate that longer continuous bouts may be more beneficial. This aligns with Sahli et al. (2022), who associated positive mood balance with lower fatigue and TMD scores in reduced intermittent play.

Our findings on the benefits of continuous bouts contrast with Sparkes et al. (2020), who observed decreased TMD and increased positive mood in intermittent SSGs. This difference might be attributed to variations in the study design and participant characteristics. The recovery aspects noted by Kellmann and Kallus (Kellmann et al., 1999) and the physiological regeneration processes (Drust et al., 2007; Gaesser et al., 1984) provide context for understanding the mood and performance changes observed in our study. However, our results suggest that for SSGs, continuous play may offer greater benefits than the intermittent approach previously recommended.

Additionally, our findings indicate that players demonstrate lower technical skill levels during medium and shorter interval bouts compared to continuous bouts. In particular, when comparing the interval format of SSGs with the same total time, we observed improvements in all technical‐tactical variables during the continuous bout. This improvement may be attributed to increased perceived satisfaction, which could potentially enhance technical‐tactical performance. This finding aligns with previous research that examined the impact of SSGs on mood and satisfaction (Berger et al., 2000; Legey et al., 2017). Analysis of the trends in our data, particularly as illustrated in Figure 1, reveals important insights for coaches designing SSG formats. The data suggest that 3v3 games may be more beneficial when the focus is on improving passing skills. This format appears to provide more opportunities for successful passes, potentially due to the reduced number of players and increased space per player. Conversely, 4v4 games seem to offer advantages when the training emphasis is on defensive aspects. The increased number of players in 4v4 games may create more opportunities for tackles and duels, thereby enhancing defensive skill development.

Regarding the specific influence of player count on technical performance, our results suggest that in the 4v4 format, there were slightly higher percentages of successful tackles and duels but lower percentages of successful passes and ball retention compared to the 3v3 format. These trends provide valuable understanding for training design, suggesting that coaches may choose between formats based on their specific technical and tactical objectives. The larger number of competitors in 4v4 introduces more uncertainty into the game, which may explain the increased likelihood of defensive actions and the potential for fewer successful passes. The consideration of both frequency and efficiency provides a more nuanced understanding of the technical demands in different SSGs formats. While the 4v4 format may offer more frequent opportunities for defensive actions, the lower efficiency might indicate a higher level of challenge. On the other hand, the higher passing efficiency in 3v3 games, despite lower frequency, suggests this format might be more conducive to developing precise passing skills. These observations can guide coaches in selecting the most appropriate format based on their specific training objectives, whether it is increasing the frequency of certain actions or improving the efficiency of particular skills.

## 5 PRACTICAL APPLICATIONS

This study demonstrates that both continuous (12 min) and intermittent durations (2 sets of 6 min) during SSGs of 4 versus 4 and 3 versus 3 lead to a reduction in psychological stress and promote a balanced mood. However, the short intermittent duration (3 sets of 4 min) does not have an impact on the decrease of negative statements caused by the POMS and does not contribute to mood balance. Therefore, it is advisable for coaches and fitness trainers to prioritize the mood state when planning soccer SSGs, as this can enhance positive mood and improve players' intrinsic motivation. From a practical standpoint, it is recommended to utilize continuous and medium intermittent durations to improve players' mood balance and foster positive psychological states. It is also crucial to exercise caution when selecting the duration of SSGs, as playing during a short intermittent duration (e.g., 3 sets of 4 min) does not influence the players' mood state and, consequently, cannot optimize performance. In terms of technical‐tactical actions, both SSGs designs studied (3 vs. 3 and 4 vs. 4) can be considered and interchangeable to enhance performance. From a practical application, coaches should choose longer bouts when programming SSGs‐based training of 4vs4 and 3vs3 to significantly improve technical performance.

## 6 LIMITATIONS AND FUTURE DIRECTIONS

While this study identified mood balance and improved technical‐tactical performance during continuous and medium‐length small‐sided games (SSGs) compared to short‐duration games, several limitations should be acknowledged. The sample included only male semiprofessional soccer players, which may limit the generalizability of the results to other groups, including different age categories (youth and senior), different levels of competition (professional, semiprofessional, and amateur), and both genders. To improve the applicability of the results, future research should include a wider range of participants to investigate how psychological responses and technical‐tactical performance vary according to age and performance level during different SSGs durations. Furthermore, it would be valuable to investigate whether these effects persist over longer periods of time and potentially influence overall seasonal performance and player development to be able to prescribe the exact dose of SSGs for improvement of mood and technical performances. Coaches should also consider external factors such as player motivation and training load to better understand their influence on player mood and subsequent performance over different SSGs durations. A high level of motivation promotes commitment and a positive mood, which leads to better concentration and resilience, resulting in improved performance. An optimal training load is important to maintain motivation and avoid fatigue. When training intensity and volume are balanced, players maintain their energy level and positive mood. We suggest integrating tools to measure motivation (e.g., self‐assessment questionnaires) and to monitor the training load via tracking devices or rating of perceived exertion (RPE) scales. This would make it possible to better quantify the influence of these variables on the results and to clarify their respective effects on the technical and psychological performance of the participants.

## 7 CONCLUSIONS

To the best of the author’s knowledge, this study is the first to demonstrate the effects of different durations of SSGs on the mood state and technical‐tactical performance of soccer players. The results of this study emphasize the importance of controlling the duration of SSGs, as significant differences in mood disturbances were found, which could potentially impact the players' technical‐tactical performance in training and competitive settings. To elaborate, the continuous (12 min) and medium intermittent durations (2 × 6 min) of both 4vs4 and 3vs3 formats yielded better results. These durations seemed to strike a balance in mood and foster positive psychological states among the players. These findings hold great significance for coaches, as they can interpret and apply them according to the specific demands of each phase of the season. Additionally, our data trends suggest that 3vs3 games may be more effective for improving passing skills, while 4vs4 games offer advantages for developing defensive skills. These findings provide valuable knowledge for coaches to tailor SSGs formats according to specific training objectives and seasonal requirements. We recommend using continuous durations of SSGs to enhance players' mood and technical qualities while strategically alternating between 3vs3 and 4vs4 formats to target specific skill development.

## Author contributions

Zouhaier Farhani, Hatem Ghouili, Anissa Bouassida, Lamia Ben Ezzeddine, and Ismail Dergaa: conception and design. Zouhaier Farhani, Mohamed Ben Aissa, Khaled Trabelsi, Noomen Guelmami, and Lamia Ben Ezzeddine: analysis and interpretation of the data. Wissem Dhahbi, Achraf Ammar, Mohamed Mansour Bouzouraa, Nizar Souissi, Ismail Dergaa, and Anissa Bouassida: drafting of the paper. Zouhaier Farhani, Hatem Ghouili, Wissem Dhahbi, Anissa Bouassida, Lamia Ben Ezzeddine, and Ismail Dergaa: revising it critically for intellectual content. All authors gave their final approval to the version that will be published.

## CONFLICT OF INTEREST STATEMENT

The authors declare that the research was conducted in the absence of any commercial or financial relationships that could be construed as a potential conflict of interest.

## PATIENT CONSENT STATEMENT

All participants were notified of the research procedures, requirements, benefits, and risks before giving informed consent.

## Data Availability

The data that support the findings of this study are openly available upon request from the corresponding author.
